# Covid, AI, and Robotics—A Neurologist's Perspective

**DOI:** 10.3389/frobt.2021.617426

**Published:** 2021-03-25

**Authors:** Syed Nizamuddin Ahmed

**Affiliations:** Division of Neurology, Department of Medicine, University of Alberta, Edmonton, AB, Canada

**Keywords:** artificial intelligence, robotics, COVID-19, telemedicine, neurology, AI, neurologist

## Abstract

Two of the major revolutions of this century are the Artificial Intelligence and Robotics. These technologies are penetrating through all disciplines and faculties at a very rapid pace. The application of these technologies in medicine, specifically in the context of Covid 19 is paramount. This article briefly reviews the commonly applied protocols in the Health Care System and provides a perspective in improving the efficiency and effectiveness of the current system. This article is not meant to provide a literature review of the current technology but rather provides a personal perspective of the author regarding what could happen in the ideal situation.

## Introduction

Despite being one of the best healthcare systems in the world, our healthcare system is still highly inefficient, suboptimal, and redundant. From the time a person enters the hospital to the time he gets discharged, inefficiencies can be noted at every nook and corner. These inefficiencies lead to poor patient care, creation of scut work for the caregivers, poor infection control, guess work in medical management, inaccuracies in test reporting, waste of resources and very poor judgement when it comes to spending the health care dollars.

If the working machinery is of poor quality, no matter how good an operator you hire, the productivity of the machines will still be as good as the machinery employed. Unfortunately, this applies to the health care system not only in Canada but around the world. Instead of changing the machinery we keep replacing the operators. That's the big reason we have not been able to revolutionize health care. All we have kept doing is presenting the same machines in newer and more attractive packaging. The machines are still the same.

### Case Study

Let me start with a story. This is not a real story but brings you very close to reality. This is the story of so many patients that I see in the hospital. Being a neurologist, I will mold the story to fit a patient coming in for a neurological condition. For the understanding of the lay public let's start with a diagnosis familiar to most people—a seizure. So, this is the story of a patient who presents to the hospital with a new onset seizure.

### Case Review

Nora's husband got woken up at 2 a.m. on July 29th, 2020 when he heard “a deep scream” and found that Nora was convulsing, foaming from her mouth and unresponsive. She appeared gray and started breathing heavily. He called 911. Emergency Medical Team (EMT) were there within 10 min and after providing urgent care took her to the nearest emergency room. Nora was awake but somewhat disoriented by the time she arrived at the emergency room. She was triaged within 10 min but waited for another 2 h before seeing a physician. She followed simple commands, but her husband said that she was still “off and not back to the baseline.” The doctor from the rural emergency room called the neurologist on call who suggested a computerized tomography (CT) scan of the head, a spinal tap and a load of 1,000 mg of intravenous Phenytoin. CT scan was done after 2 h followed by the spinal tap. It was 4 p.m. the next day before the tests results were available and reported normal. Nora had another witnessed seizure. The emergency room (ER) physician loaded her with another 500 mg of Phenytoin and transferred her to the University Hospital. Patient was seen within an hour by the casualty officer (CO). She did not seem to have any obvious seizures. Neurology resident was consulted at 11 p.m. the next day and completed the consult by midnight the following morning.

Patient was reviewed by the staff neurologist the following morning. Although she was awake and partially responsive, she was still confused and disoriented. An urgent electroencephalogram (EEG) was requested which showed that the patient was in non-convulsive status epilepticus. At this time the patient was given an extra load of Phenytoin and the intensive care unit (ICU) ICU team was consulted. EEG was still running and showed recurrent electrographic seizures. Patient was given a load of Propofol and admitted to ICU. Since there were no respiratory symptoms and the chest Xray was normal she was not isolated. Blood was sent for CBC, LFTs, COVID PCR, CRP, and electrolytes. An MRI was requested for the morning. The COVID test came back positive. The neurologist, neurology resident and five other medical house staff were asked to self-isolate for the next 14 days due to exposure to a patient who tested positive for Covid.

The next day Propofol was tapered off and Nora was alert and oriented. She stayed in the hospital for an additional 4 days. During the hospital stay she got daily CBC and electrolytes, vital signs and neurochecks were done twice a day, she had two additional chest x-rays in the context of the positive COVID. Her MRI came back normal. On a daily basis she was seen by her nurses every shift, seen by the neurology resident and staff, met personnel from the dietary service who provided the food, and was once evaluated by the physiotherapy service. She was finally discharged on August 6th with instructions about safety and driving and a follow-up by a neurologist in 3 months.

While writing the above case I have significantly simplified the course taken by a usual patient. A typical patient will go through a way more tedious process to get the above care and typically significantly more health resources are used to provide the above care. Now before I share my thoughts on how to improve the status quo I invite the reader to pause right here and write down a list of changes they would like to see in the care of this patient. I specifically suggest imagining the applications of artificial intelligence and robotics not only because of the new reality of Covid but also to better streamline the healthcare system, optimize health delivery, ensure safety of the health providers and best use of all the information collected on this patient.

## Discussion

As I look at this case and hundreds of cases that are triaged, investigated and managed all over the country on a daily basis, I ponder “if things could be different” had we used artificial intelligence, machine learning, and robotics much more wisely. Let me be more specific. We cannot improve a system if we are unable to measure the success of that system. The definition of success is a philosophical one and in general success is defined as success based on the community norms. For our healthcare system, in-part, I define success of a system based on whether the following quantitative and qualitative outcomes are achieved:

1. Reducing morbidity and mortality related to specific diagnosis.
2. Reducing healthcare costs within affordable budgets.
3. Patient and family satisfaction.
4. Sustainability of the system.
5. Reducing wait times.

A paradigm shift in health delivery requires resources, and resources can come from proper budgeting. For long term benefits we will have to start thinking about the cumulative costs over decades rather than getting overwhelmed by instantaneous expenses. In the following few paragraphs, I would like to present an alternative to the current model and propose that such a change can make our healthcare system significantly more efficient and effective. Please be mindful that due to space and time constraints, I have significantly abbreviated and simplified my suggestions and thoughts.

## Home Health Monitors/Personalized Medical Robots

Compact devices could easily be designed, where a single device monitors your blood pressure, heart rate, blood glucose, reads and reports your electrocardiogram (ECG), detects heart attacks and cardiac arrhythmias, monitors your oxygen saturation, identifies and classifies dysarthria or aphasia based on machine learning and even assesses your gait with a smart camera. The device would also be able to provide first aid instructions. This robotic device would come to you on wheels if it detects any concern linked to your wearable device. These devices will have a Bluetooth or Wi-Fi connection with your cell phone and will enable you to call 911 and immediately transfer critical medical data to the EMS who are en route to your home. This will save significant time and help EMT make proper triaging and urgent plans.

### Medical Ambulances

Getting ambulances to rural settings in time is always a challenge. This issue can be potentially resolved by creating rural ambulance sub-stations with self-driving ambulances that will carry patients to a main ambulance station staffed with humans. This will cut down the commute to the hospitals. Because of low traffic in rural communities, self- driving ambulances will be useful for patients who can be accompanied by a family member or friend.

With Covid or any other contagious disease, the safety of paramedics is essential. This can be compromised if the EMT sits too close to the patient monitoring the vitals during the ride to the hospital. Medical robots can easily monitor cardiac functions and vitals while the EMT can sit in a separate chamber avoiding exposure to any contagion.

CT scans have already been deployed in ambulances. What if the scanners are equipped with AI technology for identifying potentially salvageable tissue in stroke patients? This will significantly shorten the door to needle time, saving, and improving more lives.

### Triaging in the Emergency Rooms Using AI Technology

One of the worst nightmares of many patients in the emergency rooms is the never-ending wait time. AND the reason for the waits is lack of personnel. The question is, do we really need a human interface to triage patients in the emergency rooms? This could be very easily done by self-serving kiosks as are seen on airports for checking-in. These kiosks could be equipped with statistical learning technology to properly triage and prioritize patients and inform the attending physicians about the urgency of the situation. At the same time the specialist on call can be kept up to date about the ER visits of patients related to their fields of expertise. IMAGINE how much time can be saved if the triage monitors in the ER communicate directly to the monitors in the on-call rooms.

### When Patients Are Admitted

People/patients get hospitalized for one or combination of the following reasons: To make a diagnosis, for investigations, treatment and supportive care and placement in a safe setting. Once the patient is admitted, there can be a lot of roadblocks for timely investigations and discharge planning. The triaging of requests for investigations is subjective to some extent. Developing AI based algorithms that are reviewed by radiologists may provide a more objective way to order tests. Routine blood work these days has become random blood work. If all investigations that are ordered on a daily basis were to be justified, the ordering physicians will be in serious trouble. And this adds up to the costs of the healthcare system. There have to be AI monitored statistical protocols for ordering investigations that will ensure that appropriate investigations get carried out on a timely basis and inappropriate redundant investigations are eliminated.

In the context of infection control, robotics can play a significant role in patient care. Daily patient rounds can be easily carried out by robots with a screen that shows the doctors face to the patients. It is a short distance telemedicine. Telemedicine does not have to be distant locations. Telemedicine can be used across the hall to maintain social distancing.

### Discharge Planning

AI can play an excellent role in discharge planning. Instead of writing random clinic notes, the healthcare providers will be required to provide object-oriented notes. This information will be fed into a master database that will provide a daily update on the roadblocks to discharging a patient.

Patients who may require a long-term care or placement will be identified earlier instead at the time of discharge.

### Clinic Visits

One of the shortcomings of the clinical care system is that in this day and age humans are still involved in data collection that could be easily done otherwise. We need to have a system in place where all relevant clinical information is collected by an automated system that also uses the tools necessary to make a diagnosis and devices a treatment plan based on evidence-based medicine. Humans will play a role in ensuring that there are no shortfalls to the system. The automated system will be able to counsel patients and can even be designed to show empathy at the right time. All follow ups will be at the patient's own home if they operate a computer or to digital clinics if they require technical help. Humans can still play a role in clinical examination until that time that proven technologies also overcome this barrier.

## Applications of AI and Robotics in Current Use

Although the current use of AI and Robotics is far from ideal, there are already many frontiers which have successfully employed these technologies and are striving for an optimal model. In the following two paragraphs I will discuss some literature pertaining to this subject, briefly touching on our own work in the use of AI in diagnostic radiology.

A complex experiment always starts from a simple one. In order to identify complex pathologies in neuroimaging one has to be able to identify the normal anatomy. This was the basis of our initial experiments to segment and identify corpus callosum on the mid-sagittal sections of the brain MRI (Li et al., 2013). We subsequently worked with different classification techniques to automatically identify various epileptogenic lesions on the MRI of the brain including focal cortical dysplasia (Wang et al., 2020), cavernous malformations (Wang et al., 2018), and mesial temporal sclerosis (Wang et al., 2019). These techniques can be potentially be implemented in remote settings where there is no access to neuroradiologists for timely interpretation of the studies. The same technique can be expanded to other regions of the human body to compliment the radiologist for an expedited reporting system. Pathologists have also been using AI techniques to classify and identify cancerous cells as a pre-screen before a more thorough and meticulous review (Brinker et al., 2021).

Robotics have dramatically changed the landscape of diagnostic and therapeutic applications of stereotaxic neurosurgery. Robotic frameless neurosurgery has exponentially cut down the time employed to completing the procedure and also minimizing the contact between the surgeon and the patient as well as with the other support staff. Robotic stereotactic surgery has become a routine in many affluent north American hospitals with excellent results and in fact with improved accuracy (Dorfer et al., 2020).

Robotic telerounding employs a wheeled robot controlled by the physician. The face of the robot is visible on the robotic screen. Such robots have been employed for rounding in the Intensive Care Units and acute stroke units (Garingo et al., 2016). My vision will be to have such robots in each and every hospital, specifically in wards that deal with contagious and infectious diseases such as COVID. With the existing commercially available devices this would be an expensive undertaking. However, cheaper models can easily be manufactured on a large scale. I believe the lives of hundreds of health care workers could have been saved if such robots were already available during the COVID pandemic.

## Fantasy vs. Reality

One of the biggest impediments in progress is the idea that “it cannot be done.” If you can imagine it, you can do it. Execution only follows imagination but otherwise is merely a random motion. The kind of medical progress that we have seen in the past 50 years would have also sounded like a sci-fi movie to those before those times. MRI scanning, functional MRI, gamma knife, stroke ambulance, clot retrieval after a stroke, laser epilepsy surgery, functional brain mapping, robotic stereotactic brain surgery, monoclonal antibodies for cancer treatment, capsule endoscopy, deep brain stimulation for movement disorders, exoskeletons for paraplegics, bionics, vagas nerve stimulators for epilepsy, artificial heart, organ transplantation, cardiac defibrillator device, cardiac pacemakers, artificial plasma, plasmapheresis, treatment with IVIG are just a few of hundreds of other examples.

If self-driving cars are possible, self-driving ambulances are just a step forward. When I was in the medical school almost 30 years ago, there was not much that could be done if a person had a stroke. Just see how much things have changed. Starting from intravenous tissue plasminogen activator, to intra-arterial treatment, then from intra-arterial treatments to clot extraction. People who would have been disabled for life are now walking out of the hospital in a few days.

I ask the readers to attend to this article with an open mind that looks at the light ahead of us. If we close our eyes, all we will see is darkness and hopelessness.

## Conclusion

A well-known saying goes that if you keep doing the same thing over and over again, you cannot expect a different result. To improve our health care system during Covid 19 and once the pandemic is over, we need to implement some dramatic measures, and we need to start thinking about a workable strategy immediately. AI, robotics and telemedicine provides a unique platform to device change. To bring about a universal change we need to start thinking at the highest administrative levels with well-defined timelines and achievable milestones.

## Data Availability Statement

The original contributions presented in the study are included in the article/supplementary material, further inquiries can be directed to the corresponding author.

## Author Contributions

This article was conceived, written, and formatted by SNA.

## Conflict of Interest

The author declares that the research was conducted in the absence of any commercial or financial relationships that could be construed as a potential conflict of interest.
